# Initial Pre‐Clinical Evaluation of the Augmented Ultra‐Low Temperature Cryoablation Catheter for Ventricular Ablations

**DOI:** 10.1111/jce.70311

**Published:** 2026-03-17

**Authors:** Katia Dyrda, Atul Verma, Borislav Dinov, Thomas Fink, Tom De Potter, Santi Raffa, Martin Sirois, Marie‐Elaine Clavet‐Lanthier, Ilya Grigorov, Pedram Pourfard, Alex Babkin, Vidal Essebag

**Affiliations:** ^1^ Montreal Heart Institute Université de Montréal Montreal Quebec Canada; ^2^ McGill University Health Centre Montreal Quebec Canada; ^3^ University Hospital of Giessen, Justus Liebig University Giessen Giessen Germany; ^4^ Herz‐ und Diabeteszentrum NRW, Ruhr‐Universität Bochum Bad Oeynhausen Germany; ^5^ Cardiovascular Center AZORG Aalst Belgium; ^6^ Zentralklinik Bad Berka GmbH Bad Berka Germany; ^7^ Adagio Medical, Inc. Laguna Hills California USA

## Abstract

**Introduction:**

Ultra‐low Temperature Cryoablation (ULTC) using nitrogen cryogen near its boiling temperature of −196°C is a novel cardiac ablation modality capable of producing titratable lesions with depth exceeding 10 mm and has been recently introduced for catheter treatment of drug‐refractory ventricular tachycardia (VT). The ablative action of ULTC can be further augmented by the expansion of the cryogen within the ablation element yielding even lower ablation temperatures while optimizing mechanical handling characteristics of the catheter.

**Methods:**

The purpose of this report is to assess the feasibility and document the first pre‐clinical experience with acute endocardial lesion formation using augmented ultra‐low temperature cryoablation in healthy porcine models.

**Results:**

A modified commercially available catheter was used to create 35 augmented ULTC lesions in four acute porcine and ovine models with energy applications durations ranging from 15 to 120 s. Lesion depth and morphology were evaluated on gross necropsy and histopathology, using Masson's trichrome staining. Lesion depth ranged from median of 5.0 to median of 12.5 mm, depending on ablation duration (*p* = 0.003), with frequent transmurality and lesion morphology exhibiting consistent features across the depth range. The post‐ablation cardiac electrograms were reduced up to 89.4% in lesions with depth exceeding 10 mm.

**Conclusions:**

Augmented ULTC can be implemented using a catheter with physical handling characteristics similar to contemporary RF catheters and may allow significant reduction in ablation time compared to conventional ULTC for the equivalent lesion depth.

## 1 Introduction

**Figure 1:**
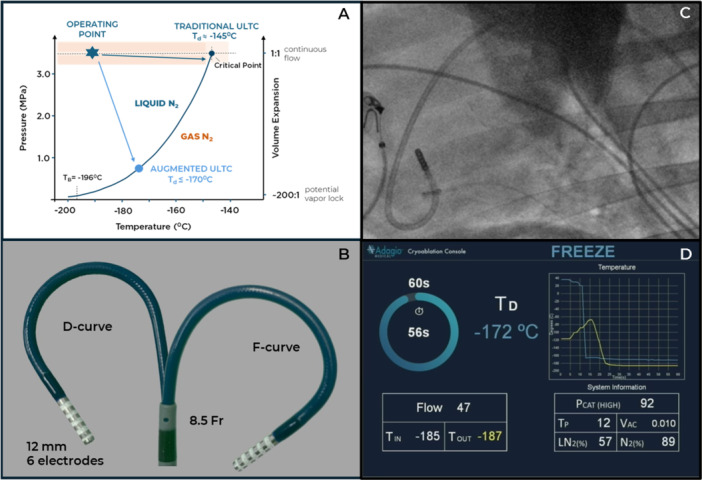
(A) Nitrogen phase diagram, showing changes to the pressure‐volume characteristic of the cryogen within the catheter with resulting ablation temperatures for both traditional and augmented ultra‐low temperature cryoablation. (B) Augmented ULTC catheter. (C) Catheter in the aortic arch for retrograde access. (D) Console screen showing rapid drop of cryoablation element temperature Td (blue line) at the initiation of the freeze.

Ultra‐low Temperature Cryoablation (ULTC) has been recently suggested for catheter treatment of atrial [1] and ventricular arrhythmias with one endocardial ablation system receiving commercial approval in Europe [2] and currently in the FDA approval trial in the USA [3]. The main benefit of ULTC in ventricular ablations is that lesion depth is titratable in animal models by the duration of energy application to values in excess of 10 mm in both healthy and scarred myocardium [4]. Such lesion depth, exceeding that of the conventional RF ablations [5] by a minimum of 2x is achievable by use of high “near‐critical” nitrogen cryogen between its boiling temperature of Tb = −196°C and it's critical temperature of Tc = −145°C. The “near critical” nitrogen combines the vapor lock‐free flow properties of the gas with the thermal capacity of the liquid [6] creating ideal conditions for deep endocardial lesion formation. The downside of the traditional ULTC was the requirement to maintain the cryogen under high pressure within the catheter resulting in a larger size (9Fr) and somewhat stiffer mechanical properties of the catheter when compared to conventional RF catheters, as well as double‐freeze ablation protocol [2, 3]. The concept of ULTC augmentation combines delivery of high‐pressure “near‐critical” nitrogen to the distal ablation element with subsequent evaporation to much lower pressure within the distal ablation element resulting in additional enthalpic cooling of the target tissue (Figure 1A) and shorter ablation‐time requirements, while lowering catheter size and stiffness. The purpose of this report is to document the first pre‐clinical experience with endocardial lesion formation using augmented ultra‐low temperature cryoablation.

## 2 Methods

Commercially‐available ventricular ULTC system (vCLAS, Adagio Medical Inc. Laguna Hills, CA), which has been described previously [2] was modified to support augmented ULTC principles, resulting in 8.5 Fr bi‐directionally deflectable (D/F‐curves) catheters with shorter (12 vs. 15 mm) ablation element, featuring six equidistant 1 mm electrodes configured in three bipoles for pacing and sensing (Figure 1B). Greater than threefold lowering of the terminal pressure within the catheter to support ULTC augmentation (Figure 1A) required minimal modification of the pressure output controls within the console hardware while providing for additional safety margin and flexibility of catheter design, including ~77% proximal‐to‐distal modulation of the catheter stiffness profile and flexibility of the catheter‐cryoablation console connection were to replicate handling behavior of conventional RF catheters. Console SW was modified to accommodate new cryogen flow dynamics and enable shorter ablation times.

Single‐freeze ablations with durations ranging from 15 to 120 s were performed in the left ventricle (LV) and right ventricle (RV) of porcine and ovine subjects at the Montreal Heart Institute (Montreal, Canada) and Orsi Laboratories (Gent, Belgium) under general anesthesia and mechanical ventilation using standard fluoroscopic guidance and (where available) electroanatomic mapping (EAM) and intracardiac echocardiography (ICE) (Ensite NavX with HD Grid and Zonaire, both Abbott Inc.). 8.5 Fr deflectable sheath (Agilis NxT, Abbott Inc) was used for transeptal and optionally for the retrograde (Figure 1C) access to LV. The contact between the catheter ablation element was ascertained by using bipolar electrograms obtained from six electrodes of the ablation element and by intracardiac echo (ICE).

Lesion dimensions were assessed at gross necropsy immediately upon termination. The subsequent histopathological analysis using Masson's trichrome staining was performed in a subset of lesions after a minimum of 5 days stabilization in 10% formalin solution to confirm gross lesion dimensions and investigate lesion morphology by a single, ablation‐protocol blinded observer. In addition, the magnitude of EGM attenuation was calculated as a ratio of peak‐to‐peak voltage from ablation catheter bipoles pre‐ and post‐ablation. Non‐parametric Mann‐Whitney and Student *t*‐test statistics were used for the analysis of continuous variables. The research protocols were approved by the Institutional Animal Care and Use Committees in the respective institutions and conformed to the appropriate regulatory guidelines.

## 3 Results

**Figure 2:**
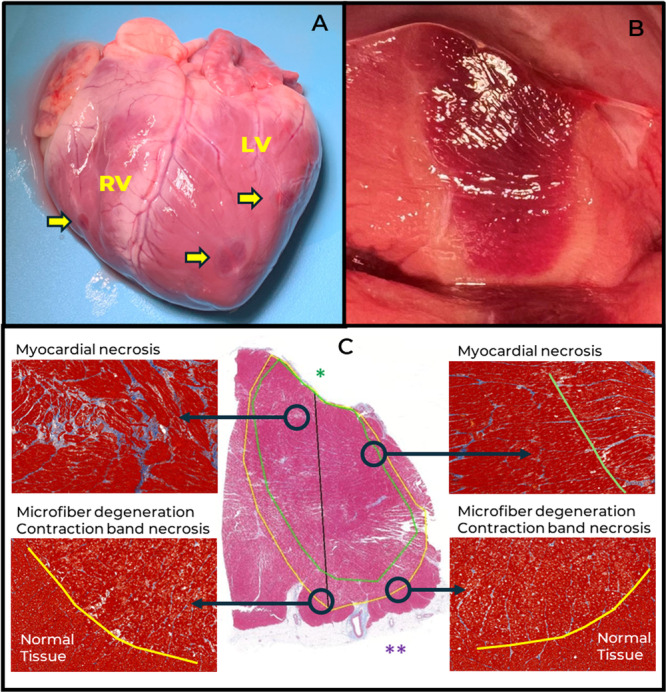
(A) Gross necropsy images of the explanted heart with transmural lesions designated with a yellow arrow. (B) Lesion cross‐section showing distinct core and rim ablation zones. (C) Lesion histologic appearance showing morphological difference between the core and rim sections of the lesion.

A total of 35 augmented ULTC ablations were performed and assessed in two (2) porcinis and two (2) ovines with weight ranging from 50 to 70 kgs, with 15 and 30 s ablations mostly confined to the RV and 60, 90, and 120 s ablations performed in the LV. The lesions were placed in multiple anatomical segments of the ventricles including septum, outflow tracts and free walls in order to minimize the probability of lesion overlap. The temperature of the cryoablation element reached Td ≈ −170°C within a few seconds of freeze initiation (Figure 1D), in line with the expected augmentation effect (Figure 1A) and substantially lower than Td ≈ −145°C reported in clinical cases of traditional ULTC [3]. Lesion transmurality was observed in 47% (9/19) of RV ablations and 25% (4/16) of LV ablations (Figure 2A). The gross appearance of the lesions consisted of the dark core surrounded by pale halo (Figure 2B). The histological analysis confirmed the presence of two distinct zones with an internal core characterized by necrosis and an outer rim representing the combination of myofiber degeneration with varying degrees of contraction band necrosis (Figure 2C). The width of the rim was approximately 1 mm and was largely independent of lesion depth and lateral dimension. The average lesion length and width were 14 and 8 mm, respectively, consistent with tissue opposition along the full length of the ablation element in most of the cases as well as lateral expansion of the ice ball, as reported previously [4].

**Figure 3:**
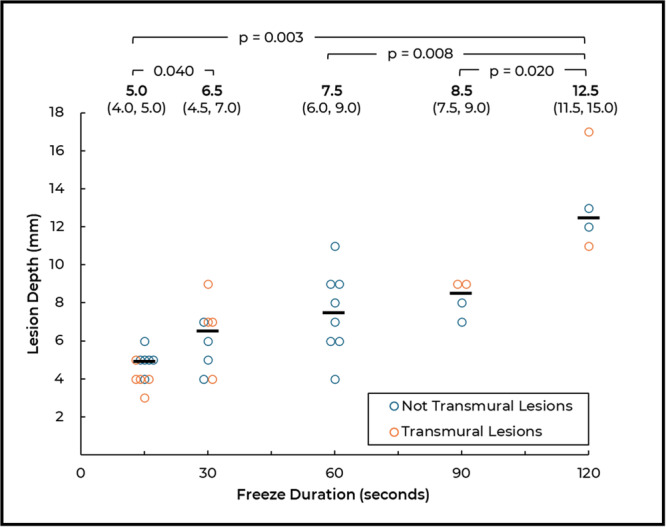
Lesion depth on gross necropsy as a function of freeze duration. Horizontal bars indicate the median lesion depth for each duration.

The gross lesion depth, measured from the endocardial surface to the edge of the outer rim (or the epicardium for transmural lesions) is shown in Figure 3, demonstrating clear depth titration effect expected of the ULTC and depth ranging from 3 to 17 mm which is consistent with clinical targets for ventricular ULTC [2]. The median depth of lesions ranged from 5.0, IQR [4.0–5.0] mm for 15 s freezes to 12.5, IQR [11.8–14.0] mm for 120 s freezes, with both the overall trend and the difference between the freeze durations obtained within each ventricle being statistically significant. Across both ventricles, there was no statistical difference between twenty‐two (22) non‐septal lesions with an average freeze duration of 54 + /− 36 s and lesion depth of 7.5 + /− 3.3 mm vs. thirteen (13) septal lesions with average duration and depth of 41 + /− 33 s and 6.2 + /− 2.4 mm, respectively. The post‐ablation EGMs were dominated by the far‐field component. The average EGM amplitude reduction across 15 lesions of varying duration was 80.9% + /−10.8%, reaching 89.4% + /− 4.3% in LV lesions with depth exceeding 10 mm. The EGM amplitude reduction in transmural lesions was 83.7% + /− 7.8% as shorter duration transmural lesions in the RV showed smaller reduction in EGMs vs. their LV counterparts (79.6% + /− 5.6% vs 91.9% + /− 2.7%).

## 4 Discussion

Dimensions and (to limited extent) morphology of the ULTC lesions in pre‐clinical models have been described before for both early experimental [7] and clinical implementations of the technology [4]. Bourier et al. [7] reported an average of 5.6 + /− 2.2 mm lesion depth after predominantly single 240 s ULTC freeze in porcine ventricles, 100% transumurality and complete elimination of the local electrograms in 96.9% of the lesions. In comparison, Dewland et al. [4] reported a median depth of 9 mm after a single vs. 12 mm after a double (“freeze‐thaw‐freeze”) 120 s ULTC applications (*p* = 0.49) in healthy and 8 vs. 13 mm (*p* = 0.061) in scarred porcine LV myocardium, with LV transmurality achieved in 0% of single and 21% of double applications, as currently implemented in clinical protocols [2, 3].

In this study, the transmurality was observed in 25% of LV lesions, but varied from 0% in applications of 60 s duration to 50% in applications of 120 s duration (Figure 3). Since the goal of the ventricular ablation is to interrupt re‐entrant circuits with critical components mostly localized to the borderline zone between the scarred and the healthy tissue at depth dependent on the etiology and the extent of the underlying disease [8], it is the titratability across the range of myocardial thicknesses representative of both RV and LV rather than notional transmurality that makes ULTC in general and augmented ULTC in particular a promising modality for VT ablations. And while the current investigation has not demonstrated extension of the maximum lesion depth by utilizing ULTC augmentation and lower ablation temperature, it is highly suggestive of the reduced ablation times required to achieve the lesions of the equivalent depths vs. conventional ULTC.

The overall acute histological findings within this study fall within the spectrum described in earlier ULTC reports. The confinement of the contraction band necrosis to the outer rim of the lesions has been previously reported for RF ablations [9] is an interesting new observation in the context of ULTC, warranting further elucidation. The 80.9% overall reduction in EGM amplitude dominated by far‐field and 89.4% reduction in lesions with depth exceeding 10 mm appears consistent with 72.4% reduction reported after a single application of pulsed field ablation (PFA) from the lattice catheter with associated lesion depth of 5.6 mm [10].

The PFA has recently gained broad acceptance for atrial fibrillation (AF) ablation, mostly on account of the decreased procedure time with non‐inferior effectiveness and safety profiles compared to RF and cryoballoon ablation [11]. The use of PFA for ventricular ablations of both premature ventricular complexes (PVC) as well as scar‐related VTs is currently in the early feasibility phase, with a number of case studies and small cohorts describing the use of the atrial PFA catheters [12, 13, 14], including in conjunction with primary RF ablations, as well as ventricle‐specific developments [15]. Reported acute and chronic observations suggests outcomes commensurate with those observed in RF ablations, along with a number of issues specific to PFA modality and use of atrial catheters, such as coronary spasm during epicardial ablation requiring nitrates, hemolysis, inability to reach desired ablation locations, inability to establish stable tissue contact, rather extended procedure times, and, in case of ventricle‐specific > 10 kV PFA, requirement for general anesthesia and paralytic agents [15]. In summary, these data suggest that the recognized advantages of the atrial PFA may not be easily translatable into VT ablations, whereas a number of clinical watchouts may be exacerbated by the significant increase in the required pulse voltage to target deep myocardial tissue and the proximity of sensitive intra‐ and extracardiac structures.

In comparison, the CRYOCURE‐VT study of 64 patients with both ischemic and non‐ischemic (22%) cardiomyopathy undergoing endocardial ULTC ablations under both general anesthesia and deep sedation (43%)^2^ demonstrated 94% acute success, 0% primary safety endpoints (including no reported coronary spasm), combined with 81% freedom from ICD shock at 6 month. The average number of lesions per patient was 9 with average freeze time 34 min, reflective mostly of double freezes of 2 min duration for an average target lesion depth of 7–8 mm. Applying the depth‐duration correlation demonstrated in Figure 3 suggests that the use of augmented ULTC in similar ablation strategy might limit the average freeze time to 1 min per patient, effectively reducing transpired ablation time by 50%–75%, matching if not exceeding the benchmarks reported in PFA [15] with the additional benefit of lesion depth titration and overall safety. The early feasibility stage of US FULCRUM‐VT study largely mimicked the results of CRYOCURE‐VT trial with slightly higher number of ablations (9) and freeze time (47 min) per patient and similar safety and effectiveness in population with 55% of non‐ischemic cardiomyopathy [3], and the larger 209‐patient has recently announced completion of the enrollment with 6‐month primary endpoints to be available in early 2026.

### 4.1 Limitations

The study represents first pre‐clinical experience with the new catheter‐based ablation modality. The main limitations of this study are relatively small number of subjects and lesions given a number of independent variables potentially affecting lesion depth, as well as the lack of chronic information on the dimensional and histological evolution of the augmented ULTC lesions, to be addressed in further investigations.

## 5 Conclusions

By lowering the terminal pressure of the near‐critical nitrogen cryogen while lowering ablation element temperature augmented ULTC can be implemented using catheter with physical and handling characteristics similar to those of the contemporary RF catheters. Similarly to conventional ULTC, augmented ULTC lesion depth can be titrated up to the complete transmurality in both RV and LV of the porcine model by modulating freezing time. A single freeze protocol using augmented ULTC may allow significant reduction in ablation times compared to conventional ULTC for the equivalent lesion depth.

## Conflicts of Interest

Dr. Dyrda has received consulting fees from Adagio Medical. Inc. Dr. Essebag has received a Fonds de recherche du Québec clinical research scholar award and consulting fees from Adagio Medical Inc. Dr. De Potter has received consulting fees from Adagio Medical Inc. Dr. Grigorov, Dr. Babkin and Mr. Poufard are employees of Adagio Medical Inc. Dr. Verma has received consulting fees from Adagio Medical Inc. The other authors declare no conflicts of interest.

## Data Availability

The authors have nothing to report.
